# Pain-Associated Clusters Among Nursing Home Residents

**DOI:** 10.1093/geroni/igaa057.660

**Published:** 2020-12-16

**Authors:** Andrea Budnick, Reinhold Kreutz, Adelheid Kuhlmey, Dagmar Dräger

**Affiliations:** Charité - Universitätsmedizin Berlin, Berlin, Berlin, Germany

## Abstract

Chronic pain is common in older adults, particularly among nursing home residents (NHR). Internationally, the reported pain prevalence among NHR ranges from 3.7% to 79.5%. At least one in two German NHR are diagnosed with pain. Unrelieved chronic pain is associated with reduced physical functioning and psychological parameters. Given the high prevalence of pain among NHR, we hypothesized that there were likely pain-associated clusters in our target group. Clustering is an opportunity to identify differences in pain management and may enable better targeted health-service delivery for professionals. There are no available data regarding pain-associated clusters (sub-groups of NHR) based on different items measuring pain. This study was performed using baseline data, and was part of a cluster-randomized controlled trial conducted in 12 nursing homes in Berlin. We assessed pain using the German Brief Pain Inventory (BPI-NHR) among 137 NHR (mean age, 83.33 years) capable of self-report. We performed hierarchical agglomerative cluster analysis to generate three clusters (naming is based on the mean value of each BPI-NHR item in each cluster): pain-relieved (46.72 %), pain-restricted (22.63 %), and severe pain (30.66 %). Body-Mass-Index (F(2,129) = 4.274, P = 0.016), Barthel-Index (F(2,133) = 3.246, P = 0.042), and appropriateness of pain medication (F(2,119) = 12.007, P = 0.000) differed between clusters. Parameters associated with an increased or decreased risk of being in a pain-diagnosed cluster will be discussed. The observed need for clinical interventions aiming at shifting from pain-diagnosed clusters to pain-relieved status will be reflected.

